# Golgin Subfamily A Member 5 Is Essential for Production of Extracellular Matrix Proteins during TGF-*β*1-Induced Periodontal Ligament-Fibroblastic Differentiation

**DOI:** 10.1155/2022/3273779

**Published:** 2022-07-16

**Authors:** Hyun-Jin Kim, Seong-Min Kim, Min-Jeong Choi, Young-Joo Jang

**Affiliations:** ^1^Department of Nanobiomedical Science & BK21 FOUR NBM Global Research Center for Regenerative Medicine, Dankook University, Cheonan 31116, Republic of Korea; ^2^Department of Oral Biochemistry, School of Dentistry, Dankook University, Cheonan 31116, Republic of Korea

## Abstract

Human periodontal ligament stem cells (hPDLSCs) can be differentiated into periodontal ligament- (PDL-) fibroblastic progenitors by treatment with low concentrations of transforming growth factor beta 1 (TGF-*β*1). Although much is known about the profibrotic effects of TGF-*β*1, the molecular mechanisms mediating the activation of fibroblasts in periodontal ligament-fibroblastic differentiation are not well known. Our study was to investigate the mechanism of the fibroblastic process in the periodontal ligament differentiation of hPDLSCs through the discovery of novel markers. One of the monoclonal antibodies previously established through decoy immunization was the anti-LG11 antibody, which recognized Golgi subfamily A member 5 (GOLGA5) as a PDL-fibroblastic progenitor-specific antigen. GOLGA5/LG11 was significantly upregulated in TGF-*β*1-induced PDL-fibroblastic progenitors and accumulated in the PDL region of the tooth root. GOLGA5 plays a role in vesicle tethering and docking between the endoplasmic reticulum and the Golgi apparatus. siRNA-mediated depletion of endogenous GOLGA5 upregulated in TGF-*β*1-induced PDL-fibroblastic progenitors resulted in downregulation of representative PDL-fibroblastic markers and upregulation of osteoblast markers. When the TGF-*β*1 signaling pathway was blocked or GOLGA5 was depleted by siRNA, the levels of extracellular matrix (ECM) proteins, such as type I collagen and fibronectin, decreased in PDL-fibroblastic progenitors. In addition, Golgi structures in the perinuclear region underwent fragmentation under these conditions. These results suggest that GOLGA5/LG11 is a PDL-fibroblastic marker with functional importance in ECM protein production and secretion, which are important processes in PDL-fibroblastic differentiation.

## 1. Introduction

The major isoform of transforming growth factor beta (TGF-*β*) is a polypeptide growth factor that has key functions in growth, development, and tissue remodeling. TGF-*β*1 promotes fibroblast activation/proliferation, maintains apoptosis-resistant fibroblasts, and activates epithelial-mesenchymal transition (EMT). Hence, TGF-*β*1 is regarded as a key inducer of fibrosis in various tissues and diseases [1–4]. For example, stimulation of epithelial cells by TGF-*β*1 promotes differentiation into *α*-SMA-expressing myofibroblasts, which is a key feature of renal fibrosis [5]. In addition, TGF-*β*1 induces myofibrogenic transdifferentiation in primary Achilles' tendon fibroblasts in rats (Cui et al., 2011) and regulates in vitro transition of human gingival fibroblasts (hGFs) to myofibroblasts (myo-hGFs) associated with the formation of actin stress fibers and focal adhesions [6]. In tumor cells, TGF-*β*1 leads to fibroblast activation as indicated by increased extracellular matrix (ECM) protein levels and enhanced contractility mediated by the expression of *α*-SMA-containing stress fibers [7]. In our previous study, activation of *β*-catenin by TGF-*β*1 caused PDL-fibroblastic differentiation of hPDLSCs, and the presence of TGF-*β*1 stimuli determined whether hPDLSCs differentiated into PDL-fibroblastic progenitors or cementoblasts. TGF-*β*1 treatment significantly increased the expression of PDL-fibroblastic markers; this increase was inhibited by treatment with SB431542, a TGF-*β* type I receptor inhibitor [8]. Although much is known about the profibrotic effects of TGF-*β*1 in many tissues, the molecular mechanisms mediating the activation of fibroblasts in PDL-fibroblastic differentiation are not well understood.

Activation and proliferation of fibroblasts by TGF-*β*1 occur in conjunction with the expression and secretion of ECM proteins, such as fibronectin and type 1 collagen. Similar to other ECM proteins, collagen is synthesized and modified in the endoplasmic reticulum (ER) transported to the ER-to-Golgi intermediate compartment (ERGIC) in a COPII-dependent manner and then transported out of the cell via the Golgi vesicle network. Procollagen is secreted into the extracellular space, where it is cleaved and assembled into fibrillar structures [9–13]. In this typical trafficking mechanism, the Golgi complex is a major processing center within the biosynthetic secretory pathway and is essential for the delivery and production of functional extracellular matrices, including collagen, fibronectin, and proteoglycans. The golgin proteins comprise a family of Golgi-localized coiled-coil proteins [14]. Their carboxy-terminus is anchored to the Golgi membrane, and their amino-terminus extends into the cytoplasm to facilitate vesicle tethering [15–18]. There are at least 11 golgins in humans. Although different golgins localize to different regions of the Golgi apparatus, their ability to tether different types of vesicles is consistent [16, 19]. Following vesicle capture, golgins cooperate with Rab GTPases, conserved oligomeric Golgi (COG) complex, and soluble N-ethylmaleimide-sensitive factor attachment protein receptors (SNAREs) to mediate membrane fusion [20, 21].

Some golgins have functions other than vesicular tethering. Golgin-160, for example, regulates the morphology and position of the Golgi (Yadav et al., 2012). In addition to tether ER-derived vesicles (Wong and Munro, 2014), Golgi matrix protein 130 (GM130) is involved in cell migration by interacting with the protein kinase STK25 and RasGRF to promote microtubule nucleation at Golgi membranes (Baschieri et al., 2014; Joachim et al., 2015; Preisinger et al., 2004; Rivero et al., 2009). GOLGA5, also known as Golgin-84, is a membrane-anchored Golgi protein with an extensive cytoplasmic coiled-coil domain. The presence of signal peptides and transmembrane domains indicates that GOLGA5 was inserted into the membrane. In addition, the Golgi targeting sequence was present within the last 51 residues of the protein, suggesting that its transmembrane domain may be involved in Golgi retention [22]. Although knockdown studies in cultured cells suggest that GOLGA5 is associated with the maintenance of Golgi tissue, it is not required for embryonic development, postnatal survival, or fertility in mouse models [23]. Structurally, GOLGA5 is similar to GOLGB1, which contains a coiled-coil cytoplasmic domain and C-terminal transmembrane domain that anchors to the Golgi membrane. Although GOLGB1 is reported to play crucial roles in craniofacial and skeletal development [24, 25], the physiological functions of GOLGA5 are unknown.

GOLGA5 has been identified as a PDL fibroblast-specific antigen by screening for antibodies against intact cells through decoy immunization. The purpose of this study was to investigate the role of GOLGA5 in fibroblast activation, an essential process in PDL-fibroblastic differentiation of hPDLSCs.

## 2. Materials and Methods

### 2.1. Cell Culture and Chemical Treatment

To primary culture of human periodontal ligament stem cells (hPDLSCs), third molars were obtained from adult patients (19–23 years of age) under guidelines approved by the IRB of the Dankook University (DKU NON2020-008) with approval of patients visiting Yonsei Wooil Dental Hospital and DKU Dental Hospital. PDL tissues were separated from the tooth and digested by 4 mg/ml dispase (Sigma-Aldrich) and mg/ml collagenase type I (Millipore) for 1 h at 37°C. Single cell suspension isolated from pulp tissue was incubated with *α*-MEM (Hyclone) containing 20% fetal bovine serum (Hyclone) and antibiotics (Lonza) at 37°C in 5% CO_2_. For differentiation, hPDLSCs were cultured in 6-well plates at a density of 4 × 10^4^ cells per well in *α*-MEM containing 5% FBS and followed cytokines or inhibitors added: 10 ng/ml TGF-*β*1 (Sino Biological) and 10 *μ*M SB431542 (TOCRIS) for the indicated time.

### 2.2. Construction of mAbs against the Intact Human Periodontal Ligament-Fibroblastic Progenitors

Production of mAbs against antigens of the intact cells was performed as previously reported with modification [26, 27]. Animal study protocol for antibody production was approved by the Institutional Animal Care and Use Committee of Dankook University. Briefly, cells were dissociated by using enzyme-free dissociation solution (Millipore), and 5 × 10^5^ cells in 30 *μ*l PBS (pH 7.4) were injected into the hind footpads of 11 female BALB/c mice for immunization. To generate a panel of hybridomas producing antibodies that bound to the PDL-fibroblastic progenitors, progenitor cells and hPDLSCs were injected into the left and right hind footpads, respectively. After eight times of repeated alternate immunization, a lymphocyte suspension from the left popliteal lymph nodes was fused to FO myeloma cells (ATCC). Hybridomas were cultured in DMEM supplemented with 20% FBS (Hyclone) and HAT component (Sigma-Aldrich), and the clonal selection was performed by enzyme-linked immunosorbent assay (ELISA) and flow cytometric analysis on the PDL-fibroblastic progenitors and hPDLSCs.

### 2.3. Immunophenotyping and Flow Cytometry

hPDLSCs dissociated by enzyme-free dissociation solution (Millipore) were incubated with proper antibodies or hybridoma supernatants in PBS containing 1% BSA on ice, followed by treatment with FITC-conjugated anti-mouse IgG (1 : 100, Santa Cruz) as the secondary antibody. Cells were analyzed by flow cytometry in FACSCalibur™ (BD Biosciences). Antibody binding affinity was analyzed by using Cell Quest and WinMDI program.

### 2.4. Antibody Isotyping and Antibody Gene Sequencing

The immunoglobulin isotype of each mAbs was determined by the Mouse Immunoglobulin Isotyping Kit (BD Pharmingen), according to the supplier's protocol. Rat anti-mouse IgGs, IgM, IgA, Ig*κ*, and Ig*λ* were used for coating multiwell plate, and hybridoma supernatant was applied into each well. The reference immunoglobulin mixtures (BD Biosciences) were used as positive controls. HRP-labeled rat anti-mouse immunoglobulin was added into each well, and the isotype signals were determined by optical density of 450 nm. For antibody gene sequencing, total RNA was extracted from hybridoma cells by Easy-spin™ Total RNA Extraction kit (Intron), and cDNA was synthesized by Maxime RT-PCR PreMix Kit (Intron). To sequence variable regions of antibody heavy and light chains, PCR primers were synthesized and used (Table 1) as described previously [28]. For heavy chain sequencing, two variable heavy chain forward primers were combined with an isotype-specific constant region reverse primer. For light chain sequencing, three *κ* variable light chain forward primers were combined with the corresponding constant region reverse primer. The PCR products were cloned into pBluescript KS(+) vector, and sequencing was proceeded.

### 2.5. Quantitative Real-Time PCR

cDNA for quantitative real-time PCR (qRT-PCR) was synthesized by using the ReverTra Ace™ qPCR RT kit (Toyobo). The qRT-PCR was performed by using the iTaq™ Universal SYBR Green Supermix (Bio-Rad) system. Used primers are listed in Table 2. The cycling parameters of qPCR were followed: 1 cycle for 30 s at 95°C, 40 cycles for 15 s at 95°C, and 1 min at 60°C. During PCR, a dissociation curve was constructed in the range of 65 to 95°C. The GAPDH was used as an internal control to normalize the variability in the target gene expression.

### 2.6. Statistical Analysis

Student's *t*-test was applied for statistical analysis using GraphPad Prism 6 program. All experiments were repeated three times. For all graphs, data are represented as mean ± SD and considered statistically significant for a *P* value less than 0.05.

### 2.7. Western Blot Analysis and Immunoprecipitation

Cells were lysed using 1% NP-40 buffer (20 mM Tris-HCl, pH 8.0, 150 mM NaCl, 2 mM EGTA, 2 mM EDTA, 1% NP-40, phosphatase/protease inhibitors). For western blot analysis, the lysates were separated on SDS-PAGE, transferred to a PVDF membrane (Millipore) and then probed with proper antibodies. For immunoprecipitation, the intact cells were labeled by EZ-Link Sulfo-NHSLC-Biotin (Thermo Scientific). Biotin-labeled cell extract was incubated with antibody, followed by pull-down with Protein G Agarose (Incospharm). The immunoprecipitants were separated SDS-PAGE, transferred to a PVDF membrane (Millipore), and then probed with streptavidin (Sigma). The antigenic molecules were visualized by using ECL Western Blotting Detection Kit (GE healthcare) on film or directly by Coomassie Brilliant Blue staining.

### 2.8. Immunohistochemistry and Immunocytochemistry

For antigen detection in tissues, human and mouse tooth was fixed in 4% paraformaldehyde at room temperature for 12~24 hrs and incubated in decalcifying solution (RapidCalTM, BBC Biochemical) for 2 days. Tissue was embedded in paraffin block and cut into 5 ~ 6 *μ*m-thick sections. Endogenous peroxidase activity was inhibited by incubation with 0.3% H_2_O_2_ in PBS for 30 min. The sections were incubated at RT for 1 h in blocking solution (5% horse serum in PBS containing 0.1% Tween 20; 0.1% PBST) and treated with the antibody at 4°C for 16 h. Then, tissues washed for 0.1% PBST and incubated with biotin-conjugated anti-mouse IgG (Vector Laboratories) at RT for 1 h. After washing, tissue sections were incubated with VECTASTAIN ABC Reagent (Vector Laboratories) at RT for 30 min and were incubated with the DAB substrate for the development of signals. Nucleus was detected by hematoxylin and eosin staining. Tissue on microscope slides was detected by the Upright FL microscope, Nikon Eclipse 80i (Nikon). To detect subcellular localization of the antigen, the immunostaining of cells was performed as previous reports [26, 27]. Briefly, cells on a coverslip were treated with 10% horse serum for blocking and then incubated with the primary antibody at 4°C for 16 hrs, followed by treatment with FITC-conjugated anti-mouse IgG (Jackson ImmunoResearch). Nuclei were detected by staining with 4,6-diamidino-2-phenylindole (DAPI). Fluorescence signals were detected under confocal microscopy (LSM510, Zeiss).

### 2.9. Antibody Information

For FACS analysis and immunohistochemistry, anti-PLAP-1 antibody was purchased from Thermo Fisher Scientific. For immunoblot analysis, anti-GOLGA5, anti-Integrin-*β*5, anti-collagen type-1 *α*1, anti-collagen type-1 *α*2, anti-fibronectin, and anti-actin antibodies were purchased from Santa Cruz Biotechnology. For immunocytochemistry, anti-GM130, anti-integrin-*β*5, anti-calnexin, and anti-vinculin antibodies were purchased from Santa Cruz Biotechnology.

### 2.10. GOLGA5 cDNA Constructs and Ectopic Expression

Full-length cDNAs of human GOLGA5 (NM_005113.3) were obtained from GenScript that was subcloned in pcDNA3.1(+). The ORF clone was tagged by DYKDDDDK in C-terminus. The flanking sequences of the cloning site and full ORF sequences were confirmed by sequence analysis. For ectopic expression of these construction, cells were transfected with DNAs using Lipofectamine® 2000 (Thermo Fisher Scientific) according to the manual provided by the manufacturer. During the differentiation for 8 ~ 9 days, DNA transfection was carried out repeatedly in every two days.

### 2.11. Gene Silencing

Small interfering RNA (siRNA) for GOLGA5 was designed and synthesized by Bioneer Corporation. 80,000 cells were used for knockdown and transfected with 1.5 *μ*g of siRNA by using Lipofectamine® RNAiMAX (Thermo Fisher Scientific) according to the manual provided by the manufacturer. Media were changed after incubation with siRNA for 5 hrs and cells were analyzed for further study after 24~48 hrs of transfection. During the differentiation for 8 ~ 9 days, siRNA transfection was carried out repeatedly in every two days.

## 3. Results

### 3.1. TGF-*β*1 Treatment Promotes Periodontal Ligament-Fibroblastic Differentiation of Human Periodontal Ligament Stem Cells (hPDLSCs)

**Figure 1:**
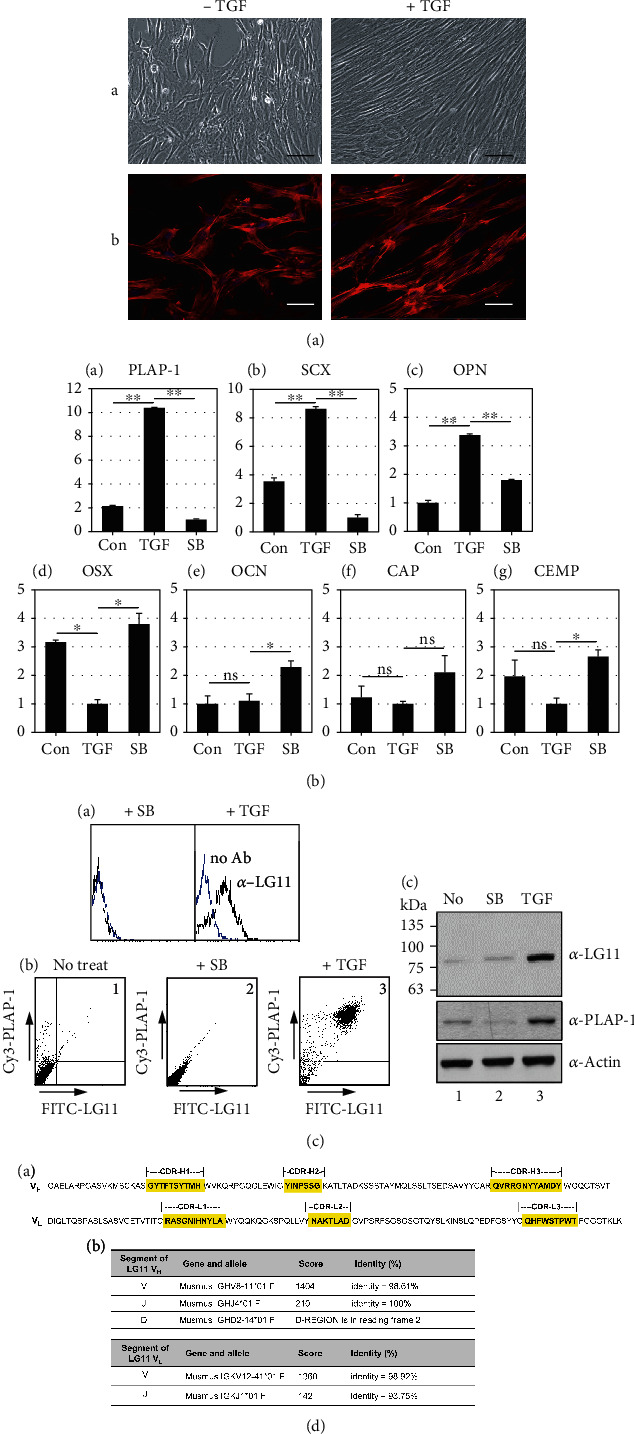
Antigenic molecule recognized by a novel anti-LG11 antibody was upregulated in the TGF-*β*1-induced fibroblastic cells. (a) Cell morphological characterization. The morphologies (A) and cytoskeletal structures (B) of hPDLSC were observed before and after TGF-*β*1 treatment. The cytoskeletal structure was shown as F-actin by phalloidin staining. (b) Evidence of successful PDL-fibroblastic differentiation derived from hPDLSCs. PDL-fibroblastic and osteo/cementoblastic marker expressions in hPDLSCs were verified by transcriptional levels. Quantitative RT-PCR analysis is performed to determine the gene expression in cells treated with TGF-*β*1 (TGF) and/or its receptor inhibitor, SB431542 (SB). Statistical significance is determined using the student's *t-*test. ns: not significant; ^∗^*P* < 0.05; ^∗∗^*P* < 0.01. (c) Antigen recognized by anti-LG11 antibody as a PDL fibroblast-specific molecule. (A) LG11 antigen specifically interacted with TGF-*β*1-induced PDL-fibroblastic progenitors (TGF), not with SB431542-treated cells (SB). (B) TGF-*β*1-induced PDL-fibroblastic progenitors were double positive of Cy3-PLAP-1 and FITC-LG11. 1: undifferentiated cells; 2: SB431542-treated hPDLSCs; 3: TGF-*β*1-induced PDL-fibroblastic progenitors. In (C), the amount of the endogenous LG11 antigen was significantly increased in total cell extract obtained from PDL-fibroblastic progenitors. (d) Sequence information and identity analysis of the variable regions of anti-LG11 antibody. (A) Amino acid sequences of mouse immunoglobulin heavy and light chain variable regions. Three complementarity determining regions in heavy and light chains (CDR-H1~3 and CDR-L1~3) were shown in shading boxes. (B) IMGT/V-QUEST database-based antibody similarity analysis.

It has been reported that low concentrations of TGF-*β*1 promote the fibroblastic differentiation of hPDLSCs [8, 29]. When hPDLSCs were treated with 10 ng/ml of TGF-*β*1 for 9 days, it can be seen that the shape of the cells took the form of a typical fibroblast that is thin and elongated, and a very regular arrangement is made so as to be directional (Figure 1(a)).

In addition to the morphological changes after 9 days of TGF-*β*1 treatment, the expression of PDL-fibroblastic markers, such as periodontal ligament-associated protein-1 (PLAP-1) and scleraxis (SCX), increased compared to that in the control (Figure 1(b), TGF in A and B). Osteopontin (OPN), a profibrogenic factor, was also induced by TGF-*β*1 treatment [30] (Figure 1(b), TGF in C). However, this increase was inhibited by treatment with SB431542 (Figure 1(b), SB in A–C). Unlike the expression of fibrogenic markers, the expression of osteo/cementogenic markers, such as osterix (OSX), osteocalcin (OCN), cementum attachment protein (CAP), and cementum protein 1 (CEMP), was either similar to that in the control group or decreased following TGF-*β*1 treatment (Figure 1(b), TGF in D–G). Treatment with a TGF-*β* type I receptor inhibitor restored their expression levels to control levels (Figure 1(b), SB in D–G). Consequently, hPDLSCs were cytodifferentiated into fibroblastic cells by treatment with TGF-*β*1 and used for further study as PDL-fibroblastic progenitors. Since the differentiation status of primary hPDLSCs not treated with TGF-*β*1 tends to be somewhat biased by age and other patient-specific features, cells treated with SB431542 were used as the control group.

### 3.2. A Novel Monoclonal Antibody, Anti-LG11 Antibody, Recognizes a PDL-Fibroblastic Progenitor-Specific Antigen of ~80 kDa

Previously, we generated a set of monoclonal antibodies against the cell surface molecules of odontoblasts using intact cells as antigens [27]. Using a strategy similar to decoy immunization, we developed a set of monoclonal antibodies against the membrane/ECM molecules of PDL-fibroblastic progenitors. hPDLSCs treated with SB431542 were used as controls. From the 20 IgG-type antibodies, anti-LG11 antibody was used to identify the specific antigen in PDL-fibroblastic progenitors in this study. Flow cytometric analysis indicated that the cell-binding affinity of anti-LG11 antibody was highly increased in hPDLSCs treated with TGF-*β*1 (Figure 1(c), +TGF in A). PDL-fibroblastic progenitors were double-positive for anti-PLAP-1 and anti-LG11 antibodies, but the cell-binding affinity of these two antibodies simultaneously disappeared after SB431542 treatment (Figure 1(c), panels 2 and 3 in B). Endogenous protein levels of LG11 antigen were notably increased in cells treated with TGF-*β*1 (Figure 1(c), lane 3 in C). These results suggest that the antigen recognized by anti-LG11 antibody may be a specific marker of PDL-fibroblastic progenitors.

In addition, we examined the expression levels according to PDL-fibroblastic differentiation time course and BMP7-induced cementoblastic differentiation [8, 31]. The GOLGA5 expression was gradually increased during TGF-*β*1-induced fibroblastic differentiation. However, this protein level in cementoblastic cells was lower than PDL-fibroblastic cells (Figure S1).

Antibody cDNA sequencing and IMGT/V-QUEST database-based antibody similarity analysis defined the complementarity-determining regions (CDRs) of anti-LG11 antibody [32, 33]. Further, amino acid sequences around the variable chains of CDRs were observed to be well conserved (Figure 1(d), A). The *V* and *J* segments of the heavy chain were found to share 98.61% and 100.0% similarity with Musmus IGHV8-11^∗^01F and Musmus IGHJ4^∗^01F, respectively. The V and J segments of the light chain were found to share 98.92% and 93.75% similarity with Musmus IGKV12-41^∗^01F and Musmus IGKJ1^∗^01F, respectively (Figure 1(d), B). These data indicate that anti-LG11 antibody is a novel antibody belonging to the IgG_1_ and IG*κ*-V12 subgroups of the light chain.

### 3.3. Anti-LG11 Antibody Specifically Recognizes Golgin Subfamily A Member 5 (GOLGA5)

**Figure 2:**
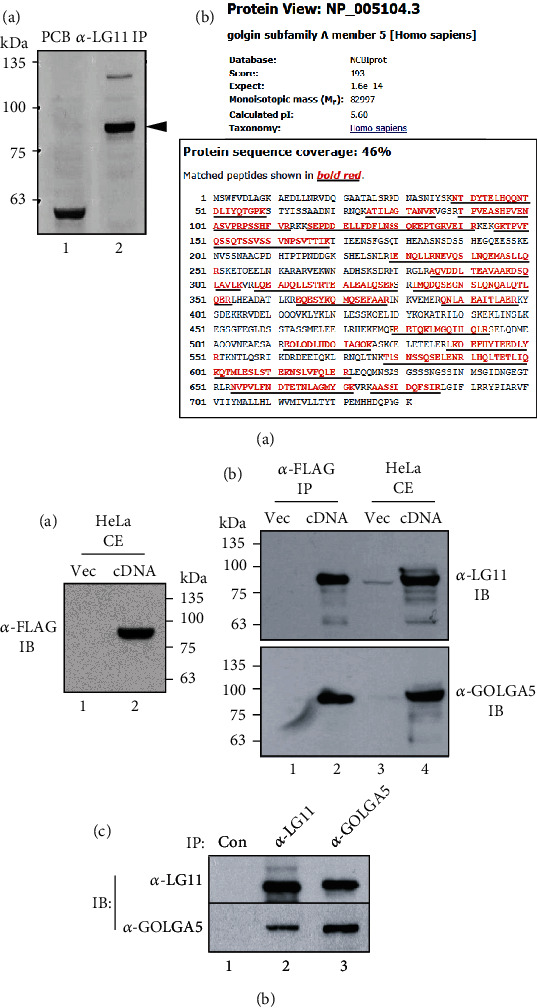
Identification of the antigenic molecule purified from the immunoprecipitates of anti-LG11 antibody. (a) The antigenic molecule recognized by anti-LG11 antibody. (A) Detection of the biotin-labeled LG11 antigenic molecule. Lane 1: preclearing without anti-LG11 antibody as control; lane 2: immunoprecipitates of anti-LG11 antibody. The antigenic molecule was indicated with arrowhead. (B) Identification of the antigenic molecule by mass spectrometric analysis. The tryptic peptides (underlines) were matched with GOLGA5 in protein sequence coverage of 46%. (b) Crossreactivity between anti-LG11 antibody and a commercial anti-GOLGA5 antibody in HeLa cells expressing GOLGA5 cDNA (A and B) and in TGF-*β*1-induced PDL fibroblastic cells (C). Full length cDNA construct tagged by FLAG was expressed in HeLa cells and detected by anti-FLAG (A), anti-LG11, and anti-GOLGA5 antibodies (B). Anti-FLAG immunoprecipitates (*α-FLAG IP*) recognized by both anti-LG11 (*α-LG11 IB*) and anti-GOLGA5 (*α-GOLGA5 IB*) antibodies. The input amount of total cell extract was indicated as *HeLa CE*. Lanes 1 and 3: vector only; lanes 2 and 4: cDNA transfection.

To detect the antigen recognized by anti-LG11 antibody, we performed immunoprecipitation using extracts from biotin-labeled PDL-fibroblastic progenitor. Protein bands between 75 and 100 kDa were strongly detected in anti-LG11 immunoprecipitates using a streptavidin-HRP-conjugated secondary antibody (Figure 2(a), arrowhead in lane 2). Tandem mass spectrometry identified GOLGA5 with 46% of protein sequence coverage (Figure 2(a), B).

**Figure 3:**
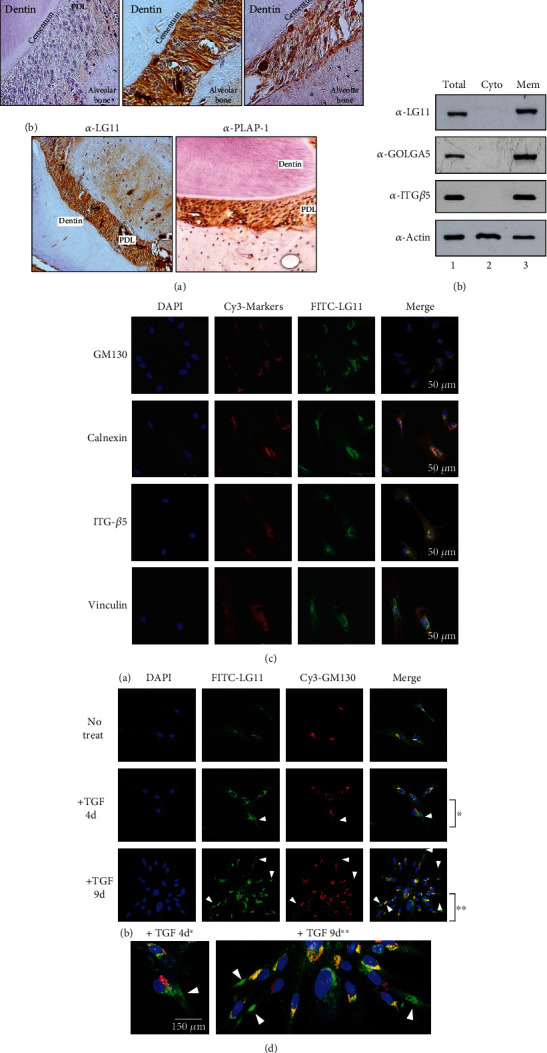
GOLGA5/LG11 is a PDL-fibroblast specific Golgi marker. (a) GOLGA5/LG11 accumulated in PDL region of tooth root. Immunohistochemistry analysis using human (A) and fetal mouse (B) tooth slices. Anti-PLAP-1 antibody was used as the positive marker of periodontal ligament. (b) GOLGA5/LG11 was detected in membrane fraction of TGF-*β*1-induced PDL-fibroblastic cells. Total: total cell extract; cyto: cytosolic phase; mem: membrane fraction. (c) Subcellular localization of endogenous GOLGA5/LG11 in undifferentiated hPDLSCs. The representative organelle markers, such as GM130, calnexin, integrin *β*5, and vinculin, were detected by Cy3 for red colors (Cy3-markers), whereas GOLGA5/LG11 was detected by FITC for green colors. Nuclei were detected by DAPI staining. (d) Subcellular localization of endogenous GOLGA5/LG11 and GM130 during TGF-*β*1-induced fibroblastic differentiation. hPDLSCs grown on coverslips were treated with TGF-*β*1 for the indicated times (4 and 9 days) and applied for immunocytochemistry. As the differentiation progressed, the more cells in which LG11 and GM130 were separated from each other were detected (arrowheads). Areas marked with asterisks (^∗^ and ^∗∗^) in (A) are enlarged and shown in (B).

The full-length cDNA construct of human GOLGA5 was cloned into a mammalian expression vector and ectopically expressed in HeLa cells. Ectopically expressed GOLGA5 could be detected by anti-FLAG antibody because this construct was tagged with a FLAG motif at the C-terminal end (Figure 3(c), lane 2 in A). Immunoprecipitates of the anti-FLAG antibody were strongly recognized by both anti-LG11 antibody and a commercial anti-GOLGA5 antibody (Santa Cruz, raised against amino acids 343-625) (Figure 2(b), lane 2 in upper and lower panels in B). Interestingly, almost no endogenous GOLGA5 protein was detected in HeLa cells (Figure 2(b), lane 3 in upper and lower panels in B), suggesting that this protein is rarely expressed in HeLa cells. In addition to ectopic GOLGA5, crossreactivity between anti-LG11 antibody and anti-GOLGA5 antibody was observed in hPDLSCs. Immunoprecipitates of the anti-LG11 antibody were strongly recognized by the anti-GOLGA5 antibody and vice versa (Figure 2(b), C). These results indicate that the LG11 antigen is identical to GOLGA5.

**Figure 4:**
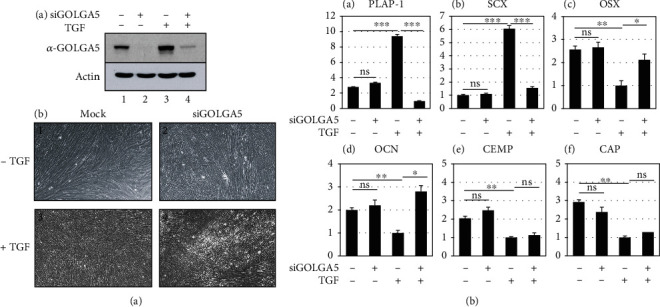
Depletion of GOLGA5/LG11 using siRNA construct inhibits TGF-*β*1-induced PDL-fibroblastic differentiation. (a) Although endogenous GOLGA5/LG11 was completely depleted (A), it was observed that cell growth was not disturbed (B). 1 and 2: undifferentiated hPDLSCs (-TGF); 3 and 4: TGF-*β*1-induced PDL-fibroblastic cells (+TGF). (b) Transcriptional expression of the representative PDL-fibroblastic and osteo/cementoblastic markers. ns: not significant; ^∗^*P* < 0.05; ^∗∗^*P* < 0.01; ^∗∗∗^*P* < 0.001. Statistical analysis was performed using Student *t*-test (*n* = 3).

Immunohistochemistry revealed that the antigen recognized by anti-LG11 antibody was abundant in the PDL region between dentin/cementum and alveolar bone in human and mouse teeth (Figure 3(a)). As expected, GOLGA5/LG11 was detected in the membrane fraction of hPDLSCs (Figure 3(b), lane 3) and colocalized with GM130, a representative Golgi marker (Figure 4(c), GM130), indicating that this protein is present in Golgi membrane. In addition to Golgi, GOLGA5 was also detected in the ER (Figure 3(c), calnexin) and the cell membrane; however, the latter location was not exactly the same as that of the integrin *β*5, ITG-*β*5 (Figure 3(c), ITG-*β*5). Interestingly, as hPDLSCs differentiated into PDL-fibroblastic progenitors, some GOLGA5/LG11 proteins did not precisely colocalize with GM130. These proteins gradually moved towards the terminal part of the long extensional region of the PDL-fibroblastic progenitors (Figure 3(d), arrowheads in A and B). These results suggest that GOLGA5/LG11 was not confined to the perinuclear region and the cis-Golgi matrix region but could migrate to outer and terminal parts of the cell during TGF-*β*1-induced PDL-fibroblastic differentiation.

### 3.4. Depletion of Endogenous GOLGA5/LG11 Inhibits the Periodontal Ligament-Fibroblastic Differentiation Potential of hPDLSCs

Endogenous GOLGA5/LG11 in undifferentiated cells and PDL-fibroblastic progenitors was depleted using siRNA constructs (Figure 4(a), lanes 2 and 4 in A). No specific morphological changes or cellular defects were detected after depletion (Figure 4(a), B). Interestingly, cells displayed no increase in the transcript levels of representative fibroblastic markers upon TGF-*β*1 treatment following GOLGA5/LG11 depletion (Figure 4(b), A and B). The OSX and OCN expression was moderately increased, while cementoblastic markers, such as CEMP and CAP, remained unchanged (Figure 4(b), C–F). The data indicate that, in Golgi apparatus, GOLGA5/LG11 plays an important role in TGF-*β*1-induced fibroblastic differentiation.

### 3.5. GOLGA5/LG11 Is Involved in the Production of ECM Proteins during Periodontal Ligament-Fibroblastic Differentiation

**Figure 5:**
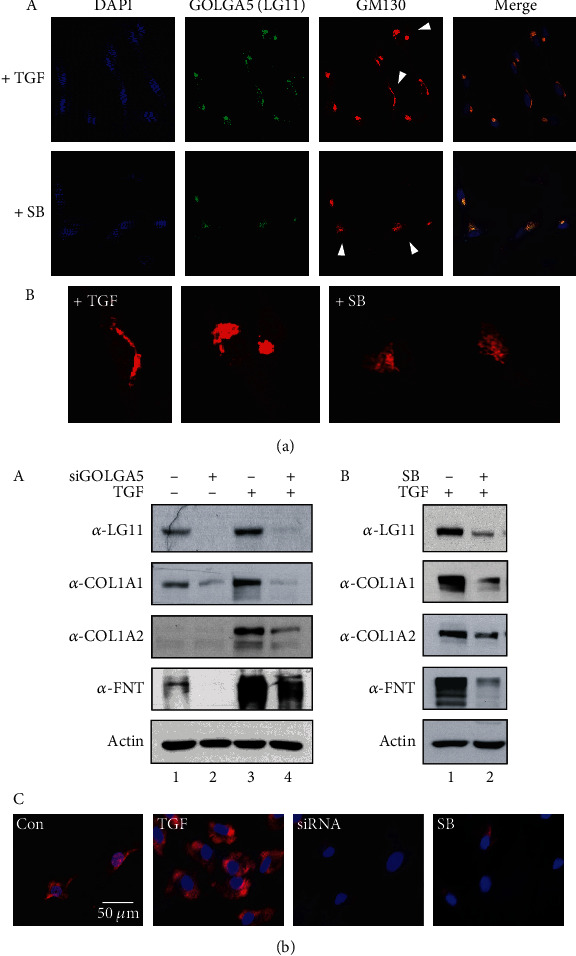
GOLGA5/LG11 is involved in synthesis of ECM proteins. (a) Inhibition of TGF-*β*1-induced fibroblastic differentiation by SB431542 treatment caused Golgi fragmentation. The structures of Golgi apparatus detected by Cy3-labeled GM130. Golgi marked with arrowheads in (A) are enlarged and shown in (B). (b) Synthesis of ECM proteins such as collagen type 1 and fibronectin in PDL-fibroblastic progenitors was significantly blocked by decrease in GOLGA5 amount, as well as GOLGA5/LG11, collagen type 1*α*1, collagen type 1*α*2, and fibronectin in fibroblastic cells were completely decreased by GOLGA5 depletion by siRNA (A) and SB431542 treatment (B). (C) Immunocytochemistry of endogenous collagen type 1. Expression of collagen type 1 in cells was detected by Cy3 signals. Nuclei were detected by DAPI staining.

To examine the structural changes in Golgi during fibroblastic differentiation, we detected endogenous Golgi proteins in hPDLSCs treated with TGF-*β*1 or SB431542 using anti-GM130 and anti-LG11 antibodies. Golgi structures developed clearly in the perinuclear region when GOLGA5/LG11 protein levels increased upon TGF-*β*1 treatment (Figure 5(a), +TGF in A and B). However, Golgi structures were fragmented and poorly organized when GOLGA5/LG11 protein levels decreased upon SB431542 treatment (Figure 5(a), +SB in A and B).

Production of ECM proteins, such as collagen and fibronectin, is essential for the fibroblastic differentiation. In a previous study, platelet-rich plasma containing TGF-*β*1 was found to stimulate fibroblastic cell proliferation and upregulate collagen synthesis in hPDLSCs [34]. Collagen synthesis occurs in the ER, followed by transport to the Golgi and then into the extracellular space where the proteins assemble for maturation. Since various modifying enzymes and chaperones are involved in this process and are recycled through Golgi retrograde transport, any mutation within the retrograde transport pathway will result in a defect in collagen production [35, 36]. We investigated the relevance of GOLGA5/LG11 in ECM protein biosynthesis, as ECMs are generally secreted via the classical method of transport from the ER to the Golgi and then to the plasma membrane. When hPDLSCs were differentiated into PDL-fibroblastic progenitors, the expression of GOLGA5/LG11 and collagen type-1 *α*1 and *α*2 significantly increased (Figure 5(b), lane 3 in A and lane 1 in B). In addition to collagen levels, fibronectin (FNT) levels were also significantly increased in fibroblastic cells (Figure 5(b), *α*-FNT in A and B). When the endogenous GOLGA5/LG11 expression was decreased by siRNA and SB431542, the expression of these ECM proteins also decreased significantly (Figure 5(b), lane 4 in A and lane 2 in B). Collagen synthesis in PDL-fibroblastic progenitors was verified by immunocytochemical analysis. Intracellular collagen levels, increased by TGF-*β*1 treatment, decreased upon depletion of GOLGA5 protein using siRNA and SB431542 (Figure 5(b), TGF, siRNA, and SB in C). These data indicate that GOLGA5/LG11 accumulates in the Golgi apparatus following TGF-*β*1 stimulation, which promotes the synthesis and secretion of ECM proteins, thereby creating an essential environment for PDL-fibroblastic differentiation of hPDLSCs.

### 3.6. Overexpression of GOLGA5/LG11 Induces Cell Death by Apoptosis

**Figure 6:**
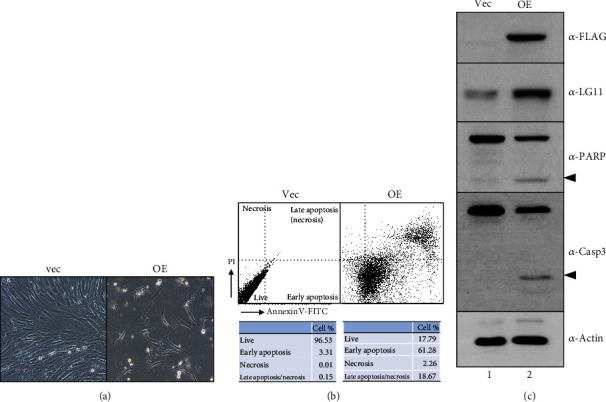
The overexpression of GOLGA5 induces the programed cell death. (a) Morphological changes of GOLGA5 overexpressed hPDLSC. (b) FACS analysis by double staining of PI and annexin V. (c) Activation of apoptotic marker proteins. The ectopic expression of GOLGA5 was detected by anti-FLAG antibody (a-FLAG). The activated fragments of PARP and caspase-3 were indicated by arrowheads. Lane 1: transfection with vector only (vec); lane 2: transfection with GOLGA5 cDNA construct (OE).

We observed that cells did not grow well and died when GOLGA5 full-length cDNA was overexpressed in hPDLSCs (Figure 6(a), OE). In addition, the proportion of annexin V-positive or PI- and annexin V double-positive cells increased approximately 23-fold from 3.46% to 79.95% (Figure 6(b)). Representative apoptotic markers, such as PARP and caspase 3, were activated by the GOLGA5 overexpression (Figure 6(c), arrowheads in lane 2). These data indicate that the overexpression of full-length GOLGA5 induced apoptosis.

## 4. Discussion

Collagen is the most abundant protein in vertebrates, accounting for approximately 90% of the organic component of bones, tendons, and ligaments. Collagen plays a key role in tissue formation provides tissues with structural support. Altered collagen biosynthesis and transport are linked to diseases such as osteogenesis imperfecta, fibrosis, and chondrodysplasia [37, 38]. Type I collagen assembles from two type I *α*1 chains together with one type I *α*2 chain, to form trimeric procollagen in the ER. Collagen precursors are transported to the Golgi apparatus within tubular structures referred to as intermediate tubules. The proalpha chains coil into a triple helix within saccules along the cis-side of the Golgi stacks. The procollagen thus produced forms bundles that give rise to cylinders accumulating on the trans-side of the Golgi stack. They are then transported to the ECM to form collagen fibrils. The mechanism of migration from the *trans*-side of the Golgi stack to the ECM has not been clearly elucidated. While considerable information is available concerning the secretion, processing, and assembly of collagen, the regulatory mechanism of collagen formation during differentiation and tissue remodeling is not understood.

We initially performed this study to identify novel membrane/cell surface markers of PDL-fibroblastic progenitors through decoy immunization. We identified GOLGA5 as the antigen recognized by a novel anti-LG11 antibody. GOLGA5, a Golgin family protein, has been proposed to participate as a transmembrane protein in intra-Golgi vesicle capture within Golgi rims during retrograde transport. GOLGA5 then gets recycled to earlier cisternae within intra-Golgi transport vesicles [17, 19].

During tissue remodeling, fibroblasts play a key role in maintaining tissue morphology, resulting in an increased deposition of ECM components, such as type I collagen and fibronectin [39]. The regulation of anterograde transport in the Golgi apparatus is vital to secrete these ECM proteins. However, our finding that GOLGA5 is a specific PDL-fibroblastic marker may explain the mechanism underlying the secretion of ECM proteins in fibroblasts from a different perspective.

Posttranslational modifications of procollagen within the ER is important for collagen maturation and depends on multiple modifying enzymes and chaperons [40]. Therefore, it can be said that retrograde protein transport promotes the recycling of enzymes and chaperons required for collagen formation. A previous report suggests that mutations in the KDEL-coupled receptor, which functions to return cargo protein to the ER, lead to collagen-deficient osteogenesis imperfecta. Hence, retrograde transport plays an important role in collagen synthesis and secretion [41]. Indeed, the synthesis of type 1 collagen and fibronectin in cells was significantly decreased upon depletion of endogenous GOLGA5 or inhibition of TGF-*β*1-induced fibroblastic differentiation (Figure 5(b)). After fibroblast activation by TGF-*β*1, there is an increase in the synthesis of ECM proteins, followed by an increase in anterograde transport. At the same time, retrograde transport also proceeds actively, thereby increasing the demand for GOLGA5 protein.

The intracellular localization of GOLGA5/LG11 was similar to that of the cis-Golgi matrix protein GM130 in the undifferentiated hPDLSCs (Figure 3(c)). However, in TGF-*β*1-induced fibroblastic differentiation, some GOLGA5/LG11 migrated towards the tip of elongated fibroblasts. The number of these cell types increased as differentiation progressed for 9 days (Figure 3(d)), suggesting that GOLGA5 may play a role in anterograde transport for direct cargo secretion in PDL-fibroblastic progenitors.

The Golgi apparatus undergoes morphological changes during normal cellular processes as well as pathological conditions such as cancer. These morphological changes result in fragmentation of the Golgi and disruption of its ribbon-like network. During cell division, Golgi fragmentation facilitates equal distribution of the Golgi into resulting daughter cells [42]. However, irreversible fragmentation of the Golgi apparatus occurs in pathological situations. Under these conditions, Golgi structural proteins are cleaved by caspases activated during apoptosis [43]. Golgi fragmentation also occurs when secretory trafficking of vesicles is perturbed [44–46]. These reports suggest that Golgi fragmentation impairs the intracellular trafficking of many proteins that are essential for biological functions.

GOLGA5 is a mitotic target that plays a key role in the assembly and maintenance of the Golgi ribbon structure in mammalian cells [47, 48]. GOLGA5 depletion results in fragmentation of the ribbon structure and reduces the volume of a normal Golgi apparatus. The transport efficiency of such Golgi apparatus is lower than that of the normal Golgi apparatus. Mice lacking GOLGA5 are viable and grow normally; however, GOLGA5 is required for embryonic development [23]. In our study, ECM protein synthesis was affected by GOLGA5 depletion in hPDLSCs (Figure 5). Further, inhibition of collagen synthesis due to defective Golgi apparatus inhibited TGF-*β*1-induced fibroblastic differentiation (Figure 4). In addition to GOLGA5, GM130, one of the most studied golgins, maintains Golgi ribbon morphology, and its defects are associated with both Golgi fragmentation and abnormal positioning [49].

In our study, cell growth was not affected by GOLGA5 depletion, but fibroblastic differentiation was inhibited (Figures 4 and 5). In contrast, the GOLGA5 overexpression induced cell defects via apoptosis (Figure 6). In a previous study, the overexpression of GOLGA5 resulted in severe fragmentation of the Golgi ribbon [47, 48], and the overexpression of GM130 caused cell pathology in a model of lysosomal storage disease and cellular defects due to the absence or malfunctioning of lysosomal enzymes needed to break down glycosaminoglycans (GAGs) [50].

In conclusion, both the gain and loss of function of GOLGA5 significantly influenced PDLSC growth and fibroblastic differentiation. We believe that GOLGA5 may be a promising marker for PDL-fibroblastic progenitors.

## 5. Conclusions

Human periodontal ligament stem cells (hPDLSCs) can be differentiated into PDL-fibroblastic progenitors by treatment with low concentrations of TGF-*β*1. Although much is known about the profibrotic effects of TGF-*β*1, the molecular mechanisms mediating the activation of fibroblasts in PDL-fibroblastic differentiation are not well understood. GOLGA5 has been identified as a PDL fibroblast-specific antigen by screening for antibodies against intact cells through decoy immunization. The purpose of this study was to investigate the role of GOLGA5 in fibroblast activation, an essential process in PDL-fibroblastic differentiation of hPDLSCs. When the TGF-*β*1 signaling pathway was blocked or GOLGA5 was depleted by siRNA, representative fibroblastic markers were downregulated, and the levels of extracellular matrix (ECM) proteins, such as type I collagen and fibronectin, decreased in PDL-fibroblastic progenitor cells. In addition, Golgi structures in the perinuclear region underwent fragmentation under these conditions. These results suggest that GOLGA5 is a ligament-fibroblastic marker with functional importance in ECM protein production and secretion, which are important processes in PDL-fibroblastic differentiation.

## Figures and Tables

**Table 1:** Primers used for RT-PCR of mouse IgG *V*_*L*_ and *V*_*H*_ gene.

Mouse heavy chain primers, *V*_*H*_ primers	
IgG	5′-ATA GAC AGA TGG GGG TGT CGT TTT GGC-3′
5′MH1	5′-SAR CTN MAG CTG SAG SAG TC-3′
5′MH2	5′-SAR GTN MAG CTG SAG SAG TCW GG-3′

Mouse kappa chain primers, *V*_*L*_ primers	
5′M*κ*	5′-GAY ATT GTG MTS ACM CAR WCT MCA-3′
Primer 6	5′-GAC ATT GTG CTG ACC CAA TCT CCA GCT TCT-3′
Primer 7	5′-GAC ATT CAG CTG ACC CAG TCT CCA-3′

Mixed base codes were indicated as follows: *R* = *a*/*g*, *Y* = *c*/*t*, *M* = *a*/*c*, *K* = *g*/*t*, *S* = *c*/*g*, *W* = *a*/*t*, *V* = *a*/*c*/*g*, and *N* = *a*/*c*/*g*/*t*.

**Table 2:** Primers used for the quantitative real-time-PCR (qPCR).

Gene		Primer sequence
Cementum protein 1 (CEMP1)	Forward Reverse	5′-GATCAGCATCCTGCTCATGTT-3′ 5′-AGCCAAATGACCCTTCCATTC-3′
Cementum attachment protein (CAP)	Forward Reverse	5′-TCCAGACATTTGCCTTGCTT-3′ 5′-TTACAGCAATAGAAAAACAGCAT-3′
Scleraxis (SCX)	Forward Reverse	5′-AGAAAGTTGAGCAAGGACC-3′ 5′-CTGTCTGTACGTCCGTCT-3′
Periodontal ligament-associated protein-1 (PLAP-1)	Forward Reverse	5′-TTGACCTCAGTCCCAACCAA-3′ 5′-TCGTTAGCTTGTTGTTGTTCAG-3′
Osteocalcin (OCN)	Forward Reverse	5′-TGAGTCCTGAGCAGCAG-3′ 5′-TCTCTTCACTACCTCGCT-3′
Osterix (OSX)	Forward Reverse	5′-GAAGGGAGTGGTGGAGCCAAAC-3′ 5′-ATTAGGGCAGTCGCAGGAGGAG-3′
Osteopontin (OPN)	Forward Reverse	5′-GTGGGAAGGACAGTTATGAA-3′ 5′-CTGACTTTGGAAAGTTCCTG-3′
GAPDH	Forward Reverse	5′-GTATGACAACAGCCTCAAGAT-3′ 5′-CCTTCCACGATACCAAAGTT-3′

## Data Availability

The data that support the findings of this study are available from the corresponding author upon reasonable request.

## Supplementary Materials

Supplementary Materials Supplementary Figure 1: the expression levels of GOLGA5 at different stages of differentiation of hPDLSCs. We examined the expression levels according to PDL-fibroblastic differentiation time course (a) and BMP7-induced cementoblastic differentiation (b). hPDLSCs were harvested every 2 days during TGF-*β*1 treatment and GOLGA5 levels were analyzed. For cementoblastic differentiation, 100 ng/ml of BMP7 was treated in cells once every 2 days for a total of 9 days. As a result, the GOLGA5 expression was gradually increased during TGF-*β*1-induced fibroblastic differentiation. As expected, this protein level in cementoblasts was lower than PDL-fibroblastic cells.
