# Supplementary material for: Golgin Subfamily A Member 5 Is Essential for Production of Extracellular Matrix Proteins during TGF-β1-Induced Periodontal Ligament-Fibroblastic Differentiation
Source: Stem Cells Int. 2022 Jul 16;2022:3273779. doi: 10.1155/2022/3273779 (PMC9308542; doi:10.1155/2022/3273779)
Supplement: Supplementary Materials — Supplementary Figure 1: the expression levels of GOLGA5 at different stages of differentiation of hPDLSCs. We examined the expression levels according to PDL-fibroblastic differentiation time course (a) and BMP7-induced cementoblastic differentiation (b). hPDLSCs were harvested every 2 days during TGF-β1 treatment and GOLGA5 levels were analyzed. For cementoblastic differentiation, 100 ng/ml of BMP7 was treated in cells once every 2 days for a total of 9 days. As a result, the GOLGA5 expression was gradually increased during TGF-β1-induced fibroblastic differentiation. As expected, this protein level in cementoblasts was lower than PDL-fibroblastic cells. [file 3273779.f1.docx]

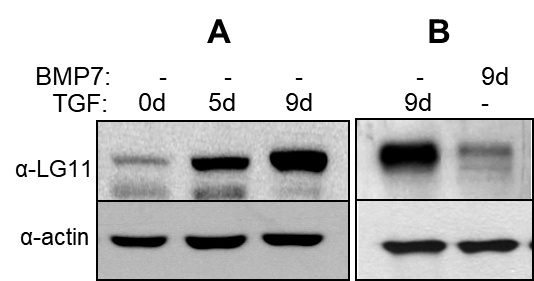


**Supplementary Figure 1.** The expression levels of GOLGA5 at different stages of differentiation of hPDLSCs. We examined the expression levels according to PDL-fibroblastic differentiation time course (A) and BMP7-induced cementoblastic differentiation (B). hPDLSCs were harvested every 2 days during TGF-β1 treatment and GOLGA5 levels were analyzed. For cementoblastic differentiation, 100 ng/ml of BMP7 was treated in cells once every 2 days for a total of 9 days. As a result, GOLGA5 expression was gradually increased during TGF-β1-induced fibroblastic differentiation. As expected, this protein level in cementoblasts was lower than PDL-fibroblastic cells.
